# Assessing sedimentation equilibrium profiles in analytical ultracentrifugation experiments on macromolecules: from simple average molecular weight analysis to molecular weight distribution and interaction analysis

**DOI:** 10.1007/s12551-016-0232-8

**Published:** 2016-11-22

**Authors:** Stephen E. Harding, Richard B. Gillis, Gary G. Adams

**Affiliations:** 1 National Centre for Macromolecular Hydrodynamics, School of Biosciences, University of Nottingham, Sutton Bonington Campus, Sutton Bonington, LE12 5RD UK; 2 School of Health Sciences, Queen’s Medical Centre, Nottingham, NG7 2HA UK

**Keywords:** SEDFIT-MSTAR, Non-ideality, Extended Fujita, COVOL

## Abstract

Molecular weights (molar masses), molecular weight distributions, dissociation constants and other interaction parameters are fundamental characteristics of proteins, nucleic acids, polysaccharides and glycoconjugates in solution. Sedimentation equilibrium analytical ultracentrifugation provides a powerful method with no supplementary immobilization, columns or membranes required. It is a particularly powerful tool when used in conjunction with its sister technique, namely sedimentation velocity. Here, we describe key approaches now available and their application to the characterization of antibodies, polysaccharides and glycoconjugates. We indicate how major complications, such as thermodynamic non-ideality, can now be routinely dealt with, thanks to a great extent to the extensive contribution of Professor Don Winzor over several decades of research.

## Introduction

Sedimentation equilibrium (SE) analysis in the analytical ultracentrifuge was introduced by Svedberg and Fåhraeus (1926) nearly a century ago as a reliable method for the assessment of the molecular weight of macromolecules in solution. Since then, the method has seen a continuous development and refinement as more and more complex systems of macromolecules have been tackled. We are grateful to the efforts of many researchers for advancing the necessary theoretical and technical approaches, and few have done more in this regard than Professor Don Winzor. Here, we assess some of the more recent developments in this field, a significant proportion of which have built on his contributions over six decades of research.

The molecular weight *M* (in Daltons), or equivalently the ‘molar mass’ (g/mol), is one of the most important parameters defining a macromolecule. SE in the analytical ultracentrifuge is a well-established method for obtaining the molecular weights of polymers (Svedberg and Pedersen 1940; Harding et al. 1992a, b) in what for many is their natural state—in solution. It has an absolute basis (not requiring calibration standards or markers, or assumptions over conformation) and has an inherent fractionation ability, without the need for columns or membranes and associated assumptions over inertness. It is also not hampered by contamination through large supramolecular particles. As such, it provides a powerful complementary probe to other methods for molecular weight analysis in solution, most notably SEC-MALS [size exclusion chromatography coupled to multi-angle (laser) light scattering], and, along with its sister technique of sedimentation velocity in the analytical ultracentrifuge, can be used to characterize a very wide range of molecular sizes from, for example, small peptides and lignins of molecular weights ∼1000 Da to huge glycoconjugate vaccine particles of molecular weights >10^8^ Da. With the use of multi-hole rotors and multi-channel cells, it is now possible to run up to 21 samples simultaneously in a single run. One drawback, which has held back its wide application, is that the procedures for data capture and analysis in the past have not been readily available, but that situation has now changed with the development of relatively easy to use analysis packages, particularly the SEDFIT platform established by P. Schuck and coworkers for the analysis of the sedimentation behavior of natural and synthetic polymer materials. Another drawback has been the complication of thermodynamic non-ideality, deriving from the large size of macromolecules and their high exclusion volumes or “molecular covolumes”. Also, since many macromolecules contain multiple charges or “polyelectrolytes” there are the additional contributions to non-ideality from polyelectrolyte repulsive effects, linked closely with the solvent environment (pH, ionic strength). The situation has been worse for SE compared to sedimentation velocity because the former generally requires high concentrations to register sufficient optical signal for analysis. Both these drawbacks have now been dealt with.

Analysis procedures start with the basic analysis of molecular weight averages (primarily the weight and *z*-averages) and also oligomeric states of assemblies using the SEDFIT-MSTAR procedure, which does not have the requirement of the assumption of a model. Then, if there is a suggestion of an interaction (self-association or interaction between a mixture of different species in, for example, protein-based systems) more advanced analysis of molecular weight distributions can be made, as recently reviewed (http://www.ncbi.nlm.nih.gov/pubmed/23377850).

Advanced interaction analysis strategies have been embodied in the multi-method analysis platform SEDPHAT (http://www.ncbi.nlm.nih.gov/pubmed/23377850). These strategies include the global fitting of many SE signal profiles acquired at different loading concentrations, different rotor speeds and different data acquisition with models that create constraints through implicit mass conservation and different interaction models, yielding binding affinities and stoichiometries (Vistica et al. 2004).

For polysaccharides and glycoconjugates which show a quasi-continuous distribution of molecular weights, SE analysis can be combined with sedimentation velocity, again within the SEDFIT platform using a procedure known as *Extended Fujita* analysis to give distributions of molecular weight. Issues of thermodynamic non-ideality can now be dealt with on a fairly routine basis, and much of the pioneering work on the interpretation of SE records where this was significant was done by Ogston, Winzor, Creeth and coworkers (see, for example, Ogston and Winzor 1975; Winzor and Wills 1986; Shearwin and Winzor 1990; Creeth and Harding 1982a; Wills et al. 1993; Wills et al. 1995; Wills et al. 1996). Thermodynamic non-ideality also affects other techniques used to measure molecular weight in solution, such as light scattering, and the relationship between the two has been established by Winzor and coworkers (Deszczynski et al. 2006; Winzor et al. 2007), who have also refined our understanding of the delicate interplay between thermodynamic and hydrodynamic (from backflow effects) factors affecting measurement of the translational diffusion coefficient using sedimentation velocity in the analytical ultracentrifuge (Scott et al. 2014).

## Sedimentation velocity vs. SE

After its invention in the 1920s the initial experiments on the Svedberg analytical ultracentrifuge were sedimentation velocity based, with early theory developed for the interpretation of photographic records from either the UV/visible absorption, Rayleigh interference or Schlieren optics systems for detecting the position and breadth of a sedimenting boundary and how this changes with time. This theory facilitated measurement of the sedimentation coefficient, *s*, from the ratio of the rate of movement of the boundary per unit centrifugal field. The sedimentation coefficient and its unit, the Svedberg, S (=10^−13^ s), became a relative measure of the size of macromolecules (7S, 11S seed globulins, etc.), although unless combined with measurement of the translational diffusion coefficient to eliminate the frictional/ shape contribution, these did not provide an absolute measure of molecular weight. The sedimentation equilibrium technique—wherein the sedimentation and diffusive forces come to equilibrium, leading to a steady-state concentration distribution not affected by frictional/shape considerations—established soon after by Svedberg and Fåhraeus (1926) provided such an absolute basis.

## Obtaining concentration distributions at SE

Sedimentation equilibrium experiments are conducted in a double sector cell, or pairs of channels in “multi-sector cells” (see Winzor and Harding 2001). One sector contains the macromolecular solution and the other the appropriate solvent. Distributions of concentration of solute *c*(*r*) versus *r* are conventionally obtained using either UV/visible absorption optics (for macromolecules with chromophores such as proteins, nucleic acids) or Rayleigh interference/refractometric optics (any macromolecule). Distributions of *c*(*r*) versus *r* can also be obtained in principle using fluorescence optics. In older or specially adapted instrumentation, Schlieren (concentration gradient) optics directly gives d*c*(*r*)/d*r* versus *r* (see Harding et al. 1992a, b). A normal prerequisite—particularly if the Rayleigh interference optical system is used—is that solutions have to be dialysed against the solvent, and the dialysate is then used in the reference sector (Winzor and Harding 2001). If the classical procedure of exhaustive dialysis is unsuitable (membrane non-inertness or, for small solutes, porosity), the macromolecular solution can be subjected to zonal gel chromatography on a column pre-equilibrated with the buffer to be used in the solvent sector. Alternatively, as a third option, the use of centrifugal ultrafiltration assemblies can achieve the same result. Creeth and Pain (1967) describe in detail the consequences of not dialyzing to constant chemical potential.

## Analysis of weight-average molecular weights: SEDFIT-MSTAR

The SEDFIT-MSTAR procedure is a model-independent SE analysis routine for obtaining primarily the weight-average molecular weight *M*_w_ for a solution of macromolecules. This includes single solute protein systems, self-associating systems, mixed systems (of, for example, different proteins, proteins + other types of macromolecule) and polydisperse systems, such as polysaccharides and many glycoconjugates. Because of thermodynamic non-ideality, the *M*_w_ returned will be an apparent value *M*_w,app_. Thermodynamic non-ideality arises from co-exclusion and polyelectrolyte effects, which, in some cases, under conditions of high dilution are not significant, but otherwise need to be taken into account. These effects can be corrected for by measuring and extrapolating 1/*M*_w,app_ to zero concentration (*c* = 0), although for many systems (small proteins) such effects are almost negligible at low concentration (∼0.5 mg/ml). SEDFIT-MSTAR *M*_w,app_ changes with local concentration *c*(*r*) in the ultracentrifuge cell and provides an estimate of the molecular weight distribution and *M*_z,app_.

It is based on the M* function (Creeth and Harding 1982b) which was originally built into a succession of programmes in FORTRAN (Harding et al. 1992a, b) and PC BASIC (Cölfen and Harding 1997), before being very recently incorporated into the SEDFIT platform of algorithms as SEDFIT-MSTAR (Schuck et al. 2014). Here, we just give a very short summary. Essentially, SEDFIT-MSTAR yields an estimate for the apparent weight-average molar mass for the whole distribution, *M*_*w,app*_ using:

1. the M* function of Creeth and Harding (1982b) defined by the integral transformation:
  1 $$ {M}^{*}(r)=\frac{c(r)-{c}_m}{k{c}_m\left({r}^2-{r_m}^2\right)+2k{\displaystyle \underset{r_m}{\overset{r}{\int }}\left(c(r)-{c}_m\right)rdr}} $$
  for sector shaped solution columns with *r* being the radial position in the ultracentrifuge cell, and the meniscus concentration *c*_*m*_ = *c*(*r = r*_*m*_). *k* is defined by:
  2 $$ k=\left(1-\overline{v}\rho \right){\omega}^2/2RT $$
  where $\overline{v}$ is the partial-specific volume, *ρ* the solvent density (Fujita 1962), ω the rotor angular velocity, *R* the gas constant and *T* the absolute temperature. M*(r) has several useful properties, the most important being the M* extrapolated to the cell base (*r = r*_*b*_) = *M*_w,app_, the apparent weight-average molecular weight for the whole distribution
  3 $$ M*\left(r={r}_b\right)={M}_{w, app} $$
2. the hinge point method: the “hinge point” in the radial distribution is the radial position at which the local concentration *c(r)* is equal to the initial cell loading concentration, *c* ^o^ (which can be evaluated from the conservation of mass equation). The SEDFIT-MSTAR algorithm evaluates the local or “point” apparent weight-average molar masses as a function of radial position, *M*_*w,app*_(*r*), and at the hinge point (*r = r*_*hinge*_)
  4 $$ {M}_{w, app}\left(r={r}_{hinge}\right)={M}_{w, app} $$
  the apparent weight-average molecular weight for the whole distribution.

**Fig. 1:**
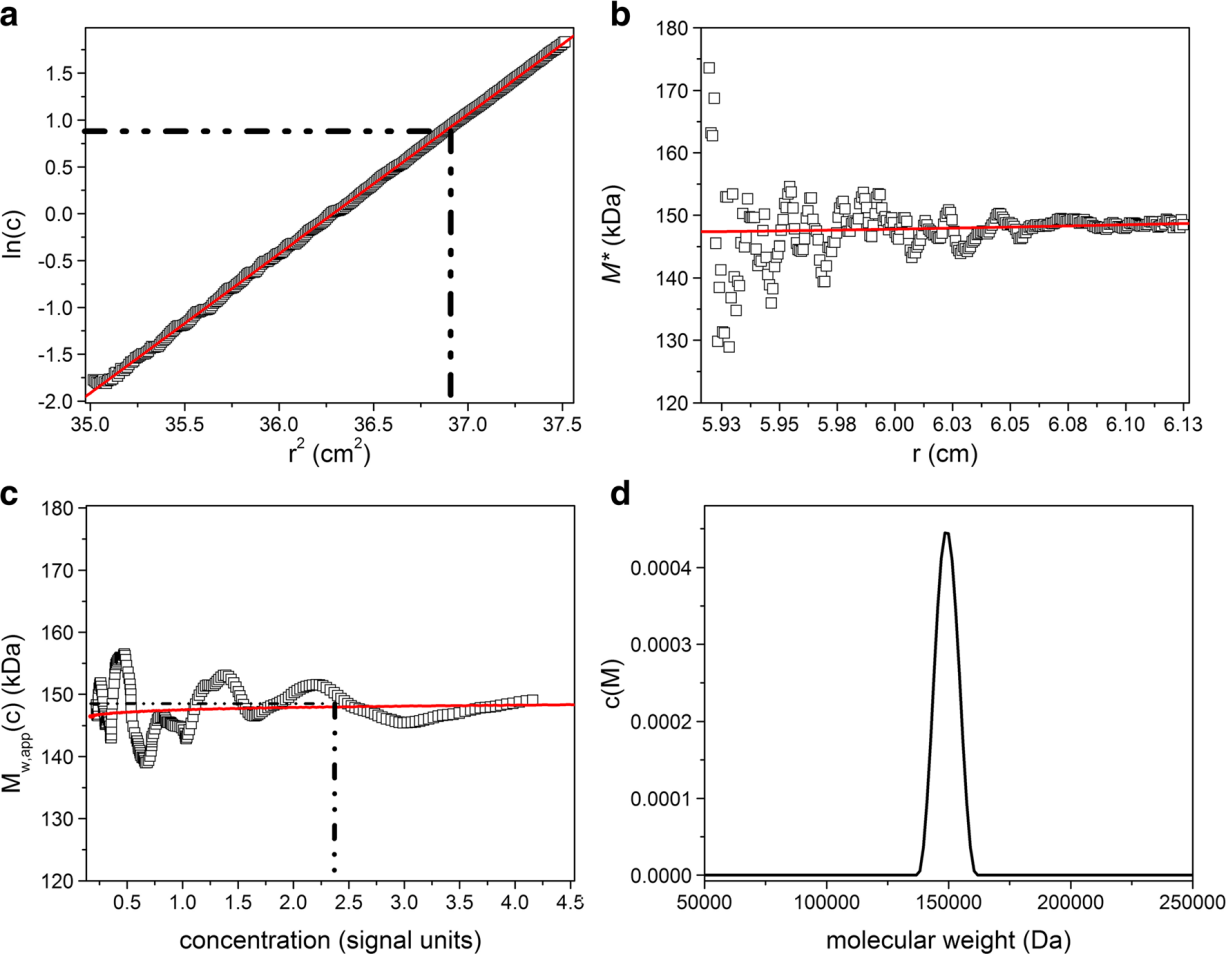
Output of the SEDFIT-MSTAR procedure for analysis on a solution of a monodisperse preparation of the human/murine hybrid antibody immunoglobulin G1 known as “Erbitux” at a loading concentration of 1 mg/ml. True *M*_w_ is ∼150,000 Da. **a** Log concentration ln*c(r))* vs. *r* ^2^ plot, where *r* is the radial distance from the centre of rotation. **b** M* versus *r* plot (*open squares*): the value of M* extrapolated to the cell base = *M*_w,app_, the apparent weight-average molecular weight for the whole distribution. **c** Point or local apparent weight-average molecular weight *M*_w_,_app_(*c*) at radial position *r* and corresponding local concentration *c(r))* plotted against the local concentration. **d** Molecular weight distribution *c(M)* vs. *M* plot. *Red line* shows the fit, *dot-dashed lines* show the position of the hinge point (**a**) and the corresponding estimation of the *M*_w,app_ value (**c**). Retrieved *M*_w_ from extrapolation of M* to the cell base = 148,000 (±2000) Da, and from the hinge point = ∼147,500 Da. Figure is from Schuck et al. (2014), with permission

**Fig. 2:**
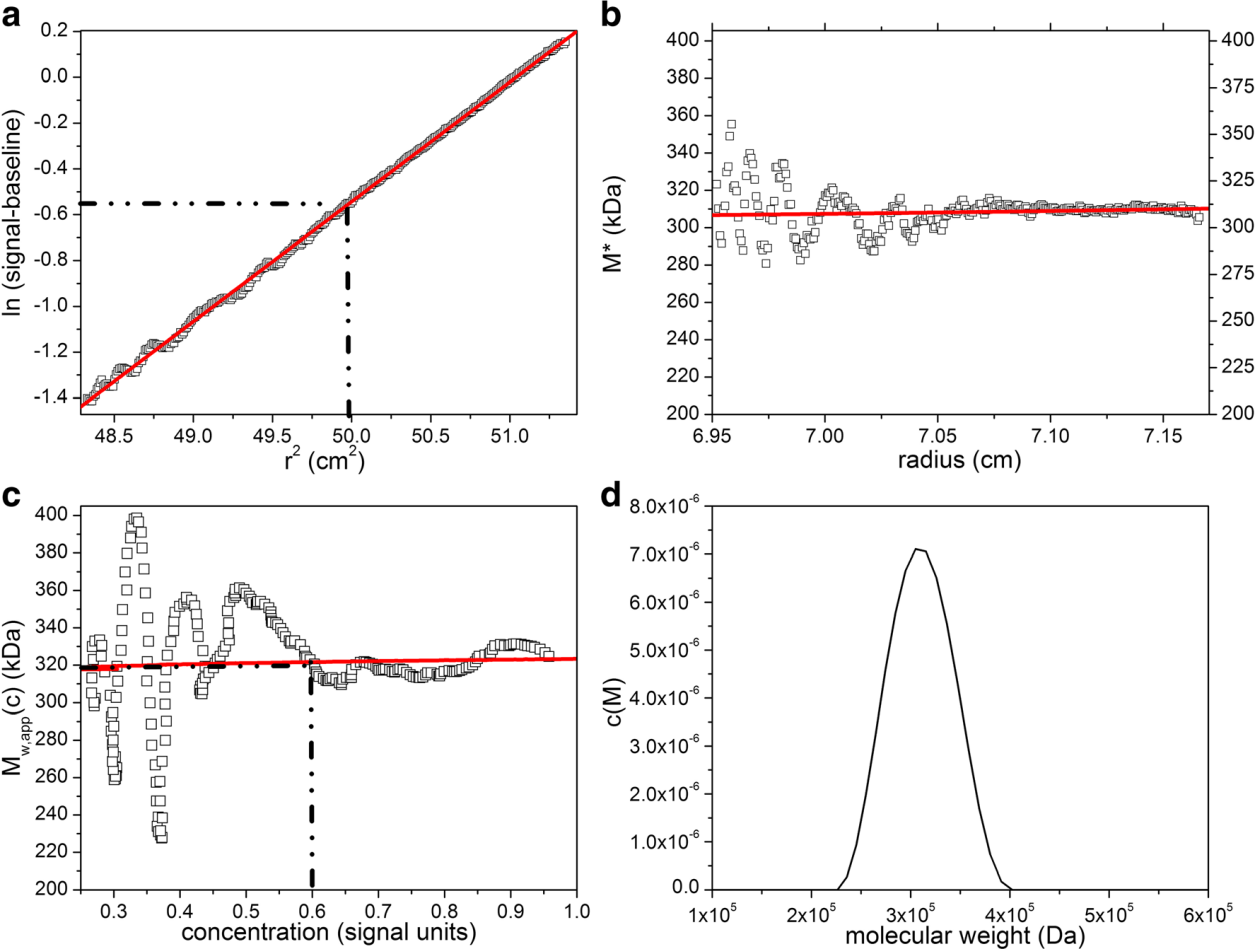
As in Fig. 1 **a**–**d** but for analysis of a polydisperse solution of the marine seaweed polysaccharide λ-carrageenan at a loading concentration of 0.3 mg/ml. **b** Retrieved value for *M*_w,app_ = 310,000 Da from extrapolation of M* to the cell base. **c** Retrieved value for *M*_w,app_ from the hinge point method ∼ 320,000 Da. Both retrieved values for *M*_w,app_ are in agreement with each other and with SEC-MALS. Figure is from Schuck et al. (2014), with permission

SEDFIT-MSTAR provides the facility for obtaining the hinge point by evaluating the initial loading concentration *c* ^*o*^ from the conservation of mass. SEDFIT-MSTAR also offers a “smart-smoothing” procedure for providing an accurate estimate for the meniscus concentration *c*_*m*_ and baseline correction, and also yields an *estimate* of the overall molar mass distribution and the *z*-average molecular weight *M*_z,app_. An example of the output from SEDFIT-MSTAR for a near-monodisperse immunoglobulin (Ig) G1 antibody is given in Fig. 1. For comparison we also give the output for a polysaccharide carrageenan (which had also been characterized by SEC-MALS) in Fig. 2. The routine also provides an estimate for the molecular weight distribution, as shown in Figs. 1 and 2. Note that even for a near-monodisperse system such as that shown in Fig. 1, the peak does not appear as a spike—rather it has some width, possibly due to the presence of trace amounts of polydispersity. This may also be indicated from the slight positive slope of the point average-molecular weight *M*_w,app_(*c*) versus local concentration *c*. A comprehensive set of other examples for synthetic and real data systems are given in Schuck et al. (2014).

Low concentrations (close to the lowest concentration limit) should be employed to minimize non-ideality effects. For a standard 12-mm path length the concentration of cells needs to be at least ∼0.5 mg/ml to give a sufficient fringe increment between cell meniscus and base (this is considerably higher than the lowest limit for sedimentation velocity experiments). The availability of cells with a 20-mm path length (Nanolytics Ltd., Potsdam, Germany) makes it possible to use a concentration as a low as ∼0.3 mg/ml, which is usually sufficient for rendering thermodynamic non-ideality contributions insignificant in many (but not all) macromolecular systems.

### Thermodynamic non-ideality

However, if working at these low loading concentrations the approximation *M*_w_ *∼ M*_w,app_ is still not valid, the conventional way of dealing with this situation is to perform a series of measurements at different loading concentration and extrapolate back to zero concentration where these effects tend to vanish. The form of the extrapolation can be linear (straight line) or non-linear (polynomial). For obtaining *M*_w,app_ using procedures that do not involve an integration there is a simple relation relating *M*_w,app_ and *M*_w_ in dilution solution:

5 $$ {M}_{w, app}={M}_w.\left\{1/\left(1+2B{M}_wc\right)\right\} $$

where *B* is the second thermodynamic virial coefficient (ml mol g^−2^). In more concentrated or non-ideal solutions additional virial terms may be necessary (*C*, *D* etc.). *M*_w,app_ values evaluated according to Eq. 4 at the hinge point conform to this relation, and a simple linear extrapolation of 1/*M*_w,app_ plotted versus loading concentration *c* yields the reciprocal of the true *M*_w_ from the intercept at *c* = 0. At higher concentrations, the extrapolation may not be linear (straight line), and an extra virial term in *c* ^2^ may be required. Furthermore, for evaluations involving an integral transformation such as Eq. 1 to obtain the whole cell distribution *M*_w_, there may also be a speed-dependent enhancement of the non-ideality effects (Fujita 1962; Harding et al. 1992a, b) leading to a larger effective value for *B* and also departure from a linear form of the extrapolation, becoming:

6 $$ {M}_{w, app}\sim {M}_w\kern.2em \hbox{--} \kern.2em 2\kern.2em Bc.{M_w}^2\left(1+{\lambda}^2{M_z}^2/12\right)+\dots $$

where *λ*, the “speed dependence parameter”, = *k*. (*r*_*b*_^2^ – *r*_*m*_^2^)/2 with *k* defined by Eq. 2.

So although *M*_w,app_ from Eq. 3 can generally be obtained to a higher precision than from the point average *M*_w,app_ evaluated from Eq. 4 at the hinge point—and without assumptions over conservation of mass—the non-ideality effect will be greater. SEDFIT-MSTAR therefore incorporates both methods of *M*_w,app_ evaluation (Schuck et al. 2014).

### A platform for further analysis

The *M*_w_ [and dependence of *M*_w_(*r*) on *c*(*r*)] obtained from SEDFIT-MSTAR, along with the corresponding sedimentation coefficient or sedimentation coefficient distribution from sedimentation velocity, may provide sufficient information about a particular system. such as its monodispersity, oligomeric state/state of aggregation, etc.. Alternatively, further processing may be required. If, on the basis of the dependence of *M*_w_(*r*) on *c*(*r*) (or *M*_w_ on different loading concentrations, *c*) and on the sedimentation velocity records there is a suggestion of a self-association or an interaction, then the *c*(*r*) versus *r* records can be further analysed to estimate interaction constants (section Interacting systems). If it is a polydisperse system like, for example, a mucin glycoprotein or a glycoconjugate vaccine, then information from SE can be used to transform a sedimentation coefficient distribution into a molecular weight distribution using the Extended Fujita algorithm (Harding et al. 2011; Gillis et al. 2013).

## Extended Fujita algorithm: combining SEDFIT-MSTAR output with sedimentation velocity to give a molecular-weight distribution

Although the SEDFIT-MSTAR algorithm can provide an approximate distribution, because of the lower speeds the resolution is quite poor. Conversely, sedimentation velocity—at higher rotor speeds—gives a much better resolution for a heterogeneous/ polydisperse system, although the distribution is (primarily) a sedimentation coefficient distribution, *g*(*s*) versus *s*. Although this is still a very useful marker of heterogeneity, the sedimentation coefficient depends not only on molecular weight but (to some extent) on macromolecular shape, and so it is desirable to convert this to a molecular weight distribution, f(*M*) versus *M*.

Fujita (1962) had originally published a method for converting a *g*(*s*) versus *s* profile to a molecular weight distribution, although his method was specifically aimed at random coil polymers. The “Extended” Fujita method (Harding et al. 2011; Gillis et al. 2013) extends the application of this method to all conformation types and has also been incorporated into the highly popular SEDFIT platform of algorithms (Harding et al. 2011; Gillis et al. 2013).

The transformation relations provided by Harding et al. (2011) are as follows:

7 $$ M={\left(s/{\upkappa}_{\mathrm{s}}\right)}^{1/b} $$

and

8 $$ \mathrm{f}(M)=\mathrm{d}s/\mathrm{d}M.\mathrm{g}(s) $$

where

9 $$ \mathrm{d}s/\mathrm{d}M=b.{\upkappa_{\mathrm{s}}}^{1/b}.{s}^{\left(b-1\right)/b} $$

**Fig. 3:**
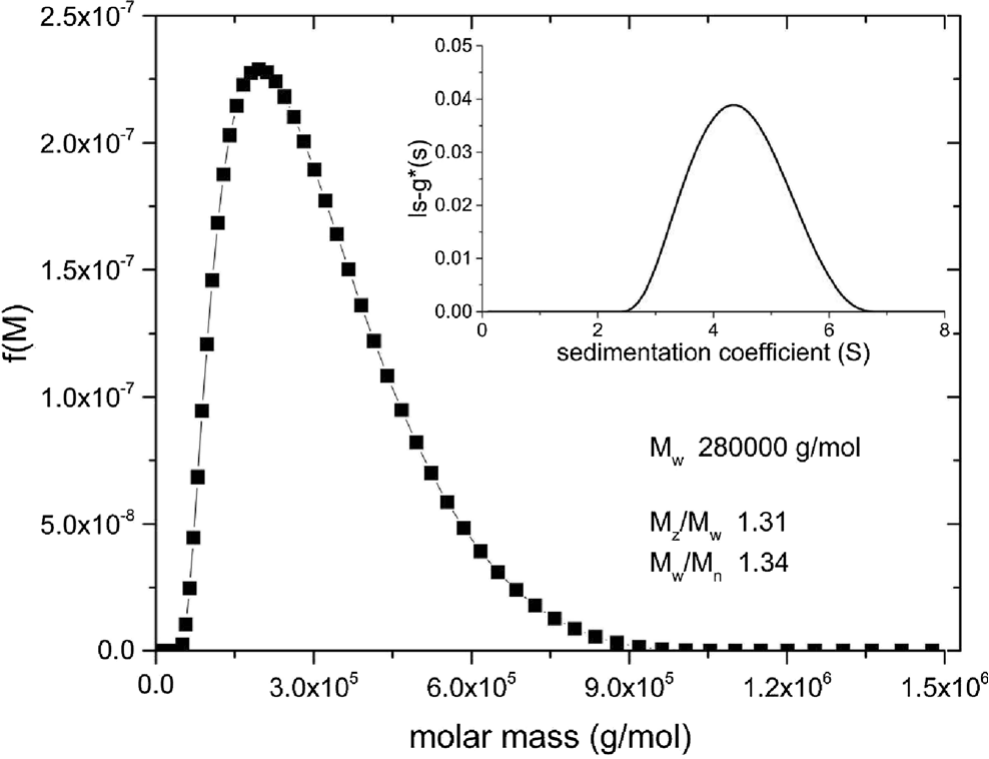
Extended Fujita method estimate of the molecular weight (expressed as molar mass, g/mol) distribution f(*M*) vs. *M* for alginate at a loading concentration of 0.03 mg/ml in 0.3 M NaCl. Transformation from a g(*s*) vs. *s* plot (*inset*) using a value for *b* of ∼0.33 (Harding et al. 2011) and κ_s_ = 0.0685, with the latter calculated from *M*_w_ = 280,000 g/mol (from SEC-MALS) and *s * = *s*_20,w_ (at 0.03 mg/ml) = 4.3S. Estimates for *M*_z_/*M*_w_ (the ratio of the z-average to the weight average molar mass) and *M*_w_/*M*_n_ (the ratio of the weight average to the number average molar mass) are also given

**Fig. 4:**
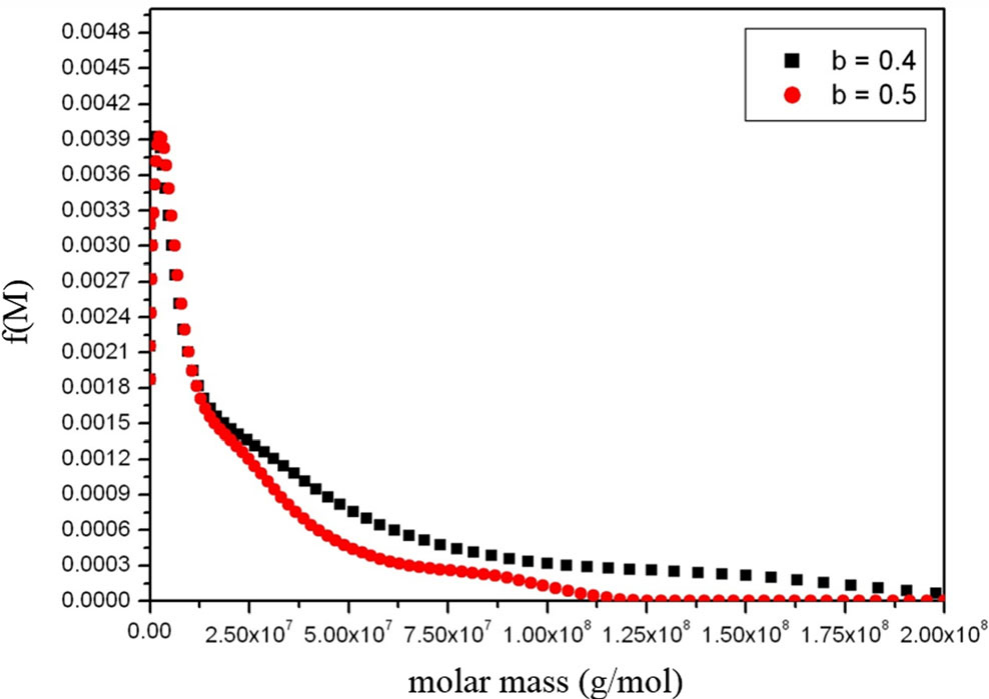
Extended Fujita method estimate of the molecular weight (expressed as molar mass, g/mol) distribution for a large glycoconjugate vaccine. The plot shows the distribution for two different values of the power law coefficient *b*

The *Extended Fujita* method needs calibrating, however, for each particular conformational system using the *b* and κ_s_ coefficients. The conformation coefficient *b* and constant κ_s_ in the transformations in Eqs. 7–9 are needed. If the conformation is known, then this will define *b*: random coils *b* ∼ 0.4–0.5; spheres *b* ∼ 0.67, rod-shaped molecules *b* ∼ 0.2. Knowledge of both the weight-average sedimentation coefficient and corresponding weight-average molar mass from a SE experiment can then be used to define κ_s_, using Eq. 7. Figure 3 gives an example of a determination for alginate at a concentration of 0.03 mg/ml. Working at low concentration also offers the additional benefit that complications through hypersharpening (larger molecular weight species being slowed down by having to sediment through solutions of the lower molecular weight species). There are two points of note:

1. The *g*(*s*) versus *s* distribution being converted should be obtained at the lowest concentration possible to minimize non-ideality. The *s* value used in Eqs. 7 and 9 should be the weight-average *s* value obtained at the same concentration as the distribution. By contrast the *M* value used to evaluate κ_s_ using Eq. 7 should *always* be the ideal value (i.e. obtained at a sufficiently lowconcentration so that non-ideality is negligible, or an extrapolated value to zero concentration).
2. If there is uncertainty in the *b* value, then at least two plausible values at the possible extremes should be tried to give an idea of the effect on the measured distribution. An example is given in Fig. 4 for a large glycoconjugate being considered for use as a vaccine. Compared with other hydrodynamic parameters, such as the intrinsic viscosity and rotational diffusion coefficients, the sedimentation coefficient is relatively insensitive to shape. Although this can cause problems with its use as a conformational probe, this helps for molecular weight distribution analysis.

## Interacting systems

**Fig. 5:**
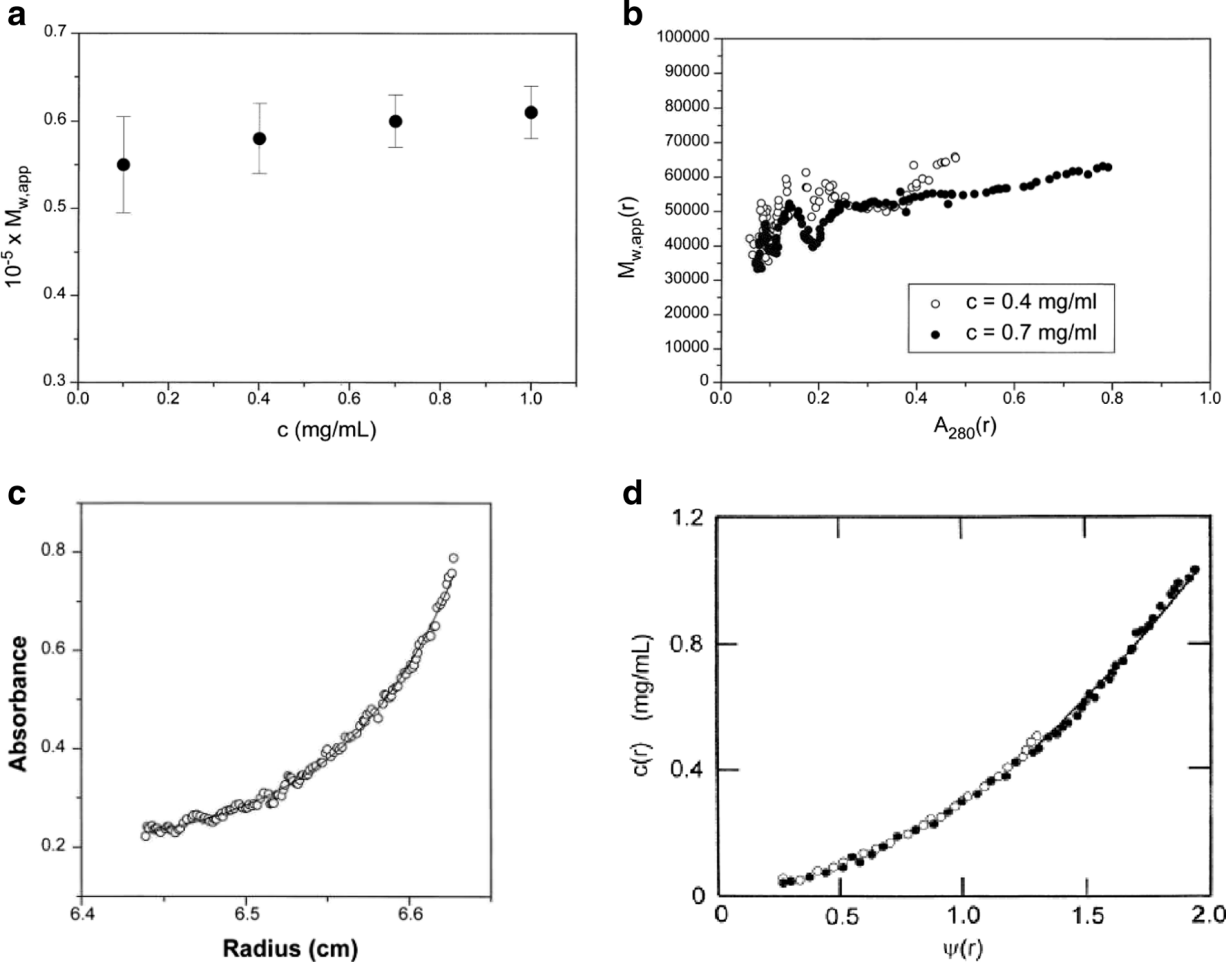
Sedimentation equilibrium analysis of the heterodimerization of the electron transfer flavoprotein ETF. The dimerization is of two equimolar components of molecular weight (*M* ) 28,900 and 33,700 Da, respectively. **a** The apparent weight-average molecular weight (*M*_w,app_) averaged over all radial positions in the ultracentrifuge cell from meniscus to cell base plotted against *c* for four different cell loading concentrations *c* showing a monomer–dimer system with a dimer molecular weight of ∼63 kDa (including FAD and AMP cofactors of collective *M* = 1120 Da) dissociating at lower concentration. **b** Plot of the ‘point’ apparent weight-average, M_w_,_app_(*r*), evaluated at individual radial positions *r* as a function of concentration [expressed as UV-absorbance A(*r*) values at 280 nm] at those radial positions. Data sets for two loading concentrations are shown. Within error, they overlap, demonstrating a reversible interaction. **c** Modelling the concentration distribution in terms of an ideal dimerization. **d** As (**c**), but in terms of the radial function ψ(*r*). The fitted data in both **c** and **d** correspond to a K_d_ ∼ (1.5 ± 0.1) × 10^−6^ M, a strong interaction. Again, the overlap at two different loading concentrations is commensurate with a reversible association. Figure is from Cölfen et al. (1997), with kind permission of the *European Biophysics Journal* (Springer Science + Business Media)

In the case of a (reversibly) interacting system, the concentration distribution in the ultracentrifuge cell, *c*(*r*) versus *r* will not only depend on molecular weight *M*, but also on mass action parameters, such as the equilibrium association constant K_a_, or the equivalent dissociation constant K_d_ (usually expressed in molar units). The distribution will also, as above, be influenced by thermodynamic non-ideality, so

10 $$ c(r)=f\left({M}_{\mathrm{b}},B,\mathrm{K},r\right) $$

where *B* is the second thermodynamic virial coefficient as described above. K represents either K_d_ or K_a_, whichever is the more convenient:

11 $$ {\mathrm{K}}_{\mathrm{a}}\left(\mathrm{l}/\mathrm{mol}\right)=\left[\mathrm{A}\mathrm{B}\right]/\left(\left[\mathrm{A}\right]\cdotp \left[\mathrm{B}\right]\right) $$

and K_d_ (mol/l) = 1/K_a_. For a monomer–dimer equilibrium, for example, correct to first order in concentration—and if virial terms higher than the second order are ignored and a binomial approximation to incorporate the contribution of K_a_ is accepted, then the relationship between the apparent molecular weight as estimated by SE and the total solute concentration *c* can be approximated by:

12 $$ \left(1/{M}_{\mathrm{w}}{,}_{\mathrm{a}\mathrm{pp}}\right)\approx \left(1/{M}_1\right)+2\left({B}_{11}-\left[{\mathrm{K}}_{\mathrm{a}}/{M_1}^2\right]\right)c $$

where *M*_1_ is the monomer molecular weight, *B*_11_ is the monomer–monomer second virial coefficient, *c* (g/ml) as above is the total solute concentration (of monomer and dimer) and the distribution of mass between these two species is given by the Law of Mass Action. The *c*(*r*) against *r* distribution (Eq. 10) at SE has historically been presented in several exponential- or logarithmic-based forms (for a review, see Creeth and Pain 1967). One popular exponential form has been given in the widely used NONLIN software (Johnson et al. 1981), which facilitates the estimation of the parameters in Eq. 10 by means of non-linear fitting algorithms. It should be noted that only one, but never both, of the thermodynamic (*B* or *BM*) and mass-interactive (K_a_) terms can be floated in a single fit. As an example, we have chosen the application of this approach to a strong interaction of the A + B = AB type, the electron-transfer flavoprotein heterodimer ETF, where the association is between one polypeptide chain of *M* ∼ 29 kDa and another polypeptide chain of 34 kDa (Cölfen et al. 1997, Wilson et al. 1997). At the low concentrations employed to study it, one can come to a reasonable approximation assuming the system to be ideal: *B*_11_ ∼ 0 in Eq. 12. For this type of system, an old but valid approach is to define the average molecular mass as a function of concentration, studied over a range in *c* where the last mentioned assumption remains valid. SE here was performed at four different loading concentrations and solute distributions recorded using UV-absorption optics. First, *M*_w,app_ was measured for each of the four concentrations using MSTAR (Fig. 5a); plots of *M*_w_,_app_ against *c* are useful for defining the stoichiometry of the system, in this case clearly a simple A + B = AB system. Then, *M*_w,app_(*r*) was plotted against the local concentrations *c*(*r*) in the ultracentrifuge cell on the same axes for different loading concentrations. If the system is a genuine reversible equilibrium, then these data sets should overlay and fall on the same *M*_w_,_app_(*r*) against *c*(*r*) curve (Roark and Yphantis 1969); this was indeed the case for the ETF system (Fig. 5b). Finally, one can fit the *c*(*r*) against *r* data sets to Eq. 10 (Fig. 5c), or an equivalent form of this in a procedure known as PSI analysis developed by Winzor and coworkers (Wills et al. 1996), to estimate K_a_ (or K_d_) (Fig. 5d). This demonstration of a strong reversible A + B interaction proved consistent with the subsequent model of the system based on high-resolution measurements (Leys et al. 2003).

For weaker interactions, the contribution of thermodynamic non-ideality effects cannot be ignored. A way of dealing with this problem was introduced by Harding, Winzor and co-workers using a procedure known as COVOL (Harding et al. 1998, 1999). It is based on earlier theory (Rallison and Harding 1985) that allows the calculation of the exclusion volume contribution to the second thermodynamic virial coefficient *B*_ex_. To do this, an estimate of the triaxial shape of the monomeric species is required (from, for example, X-ray crystallography; Taylor et al. 1983), together with the molecular weight. For calculation of the charge or polyelectrolyte contribution to the second thermodynamic virial coefficient *B*_z_, knowledge of the valency of the protein under the solvent conditions and the ionic strength of the solvent is required: *B*_11_ can then be defined as

13 $$ {B}_{11}={B}_{\mathrm{ex}}+{B}_{\mathrm{z}} $$

From the calculations, *B*_11_ can be evaluated and is no longer a variable in Eq. 12 for the analysis of K_a_ or K_d_

**Fig. 6:**
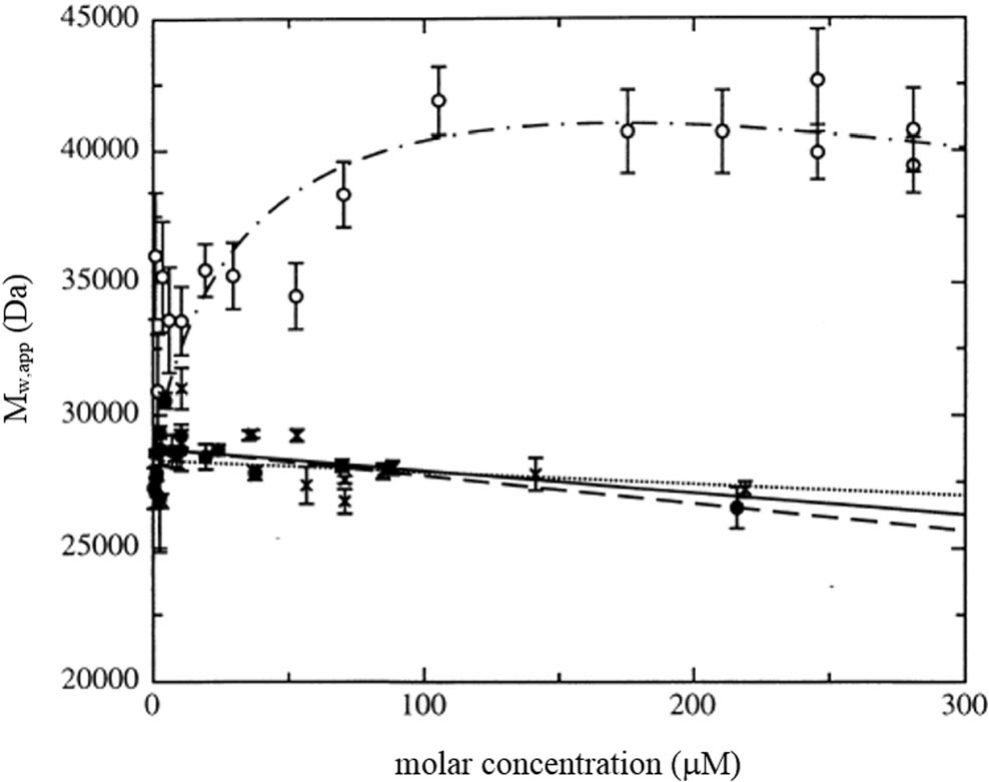
Apparent weight-average molecular weight (*M*_*w,app*_) of the CD2 and CD48 proteins and of the CD2–CD48 heterodimer as determined using sedimentation equilibrium × CD2, ● CD48 and O the CD2–CD48 heterodimer. Non-linear least-square fits to data for CD2 (*dotted line*) and CD48 (*dashed line*). *Continuous line* Predicted regression for a value of 2*BM* (from COVOL) of 10.4 ml/g. *Dotted–dashed line* Fit to CD2–CD48 heterodimer data. Using the COVOL value of 2*BM* = 10.4 ml/g, a value for the dissociation constant K_d_ ∼ (1.0 ± 0.3) × 10^−4^ M is obtained, a weak interaction. Figure is from Silkowski et al. (1997), with kind permission of the *European Biophysics Journal* (Springer Science + Business Media)

The example shown in Fig. 6 (Silkowski et al. 1997) is again for a heterologous dimerization between molecules of similar molecular mass, in this case involving two proteins involved in molecular recognition at the cell surface: CD2 (*M* = 28.3 kDa) and CD48 (*M* = 28.7 kDa). A value for the second virial coefficient (*B*_11_) based on the dimensions from X-ray crystallography of 8.5 × 2.3 × 2.5 nm for the protein CD2 and 9.4 × 4.9 × 6.7 nm for the protein CD48 and the application of the software COVOL (Harding et al. 1998, 1999) (https://www.nottingham.ac.uk/ncmh/software/software.aspx) yielded an average *B*_11_ = 1.8 × 10^−4^ ml mol g^−2^. Hence knowing this, from the experimental data, a value for K_d_ of ∼ (1.0 ± 0.3) × 10^−4^ mol l^−1^ was estimated, in good agreement with an estimate of ∼(7.5 ± 1.5) × 10^−5^ mol l^−1^ from surface plasmon resonance. In general, cases such as this, where the second virial term can be either computed or estimated, the use of software such as SEDPHAT (Vistica et al. 2004)—which facilitates the simultaneous consideration of different data sets of *c*(*r*) versus *r* obtained at different rotor speeds and temperature provides a simple and reliable way of securing a value for K_d_ and for understanding, including by “bootstrapping”, the likely levels of error present in the estimate made.

## Concluding remarks

Sedimentation equilibrium, facilitated by modern computer analysis, continues to provide a vital, matrix-free tool for characterizing the molecular weight/oligomeric state, molecular weight distribution and interaction parameters for a wide range of macromolecular systems. Although the application of analysis procedures is becoming easier, attention to detail and awareness of the complications—most notably due to thermodynamic non-ideality—remains crucial, and for this researchers are indebted to the important underpinning and authoritative work provided by Professor Winzor and colleagues over many decades.
